# A Soluble Porous Coordination Polymer for Fluorescence Sensing of Explosives and Toxic Anions under Homogeneous Environment

**DOI:** 10.3390/s23249719

**Published:** 2023-12-09

**Authors:** Jiang Jiang, Zi-Wei Li, Zhao-Feng Wu, Xiao-Ying Huang

**Affiliations:** 1 College of Life Sciences, Fujian Agriculture and Forestry University, Fuzhou 350002, China; jiang@fafu.edu.cn; 2 State Key Laboratory of Structural Chemistry, Fujian Institute of Research on the Structure of Matter, The Chinese Academy of Sciences, Fuzhou 350002, China; liziwei@fjirsm.ac.cn (Z.-W.L.); xyhuang@fjirsm.ac.cn (X.-Y.H.)

**Keywords:** fluorescence sensor, homogeneous environment, soluble coordination polymer

## Abstract

In the past decades, porous coordination polymers (PCPs) based fluorescent (FL) sensors have received intense attention due to their promising applications. In this work, a soluble Zn-PCP is presented as a sensitive probe towards explosive molecules, chromate, and dichromate ions. In former reports, PCP sensors were usually ground into fine powders and then dispersed in solvents to form FL emulsion for sensing applications. However, their insoluble characters would cause the sensing accuracy which is prone to interference from environmental effects. While in this work, the as-made PCP could be directly soluble in organic solvents to form a clear solution with bright blue emission, representing the first soluble PCP based fluorescence sensor to probe explosive molecules under a homogeneous environment. Moreover, the FL PCP solution also shows sensitive detection behaviors towards the toxic anions of CrO_4_^2−^ and Cr_2_O_7_^2−^, which exhibit a good linear relationship between the fluorescence intensity of Zn-PCP and the concentrations of both analytes. This work provides a reference for designing task-specific PCP sensors utilized under a homogeneous environment.

## 1. Introduction

The past two decades have witnessed the great progress made in fluorescent porous coordination polymers (FL-PCPs) and their applications in lighting, sensing, imaging, and so forth [1,2,3,4,5]. Thus far, most of the reported fluorescence PCPs are constructed from rare-earth metal ions due to the bright emission and abundant emission colors [6,7,8,9]. However, the rare-earth metal resource is limited and has been widely utilized in other strategic industries; thus, it is desirable to develop FL PCPs on basis of other abundant metals. On the other hand, the fluorescence detecting processes are prone to interference from environmental effects, e.g., the concentration of FL materials, excitation intensity, temperature, instrumental parameters, and so forth [10,11,12,13]. Therefore, it is still challenging to design novel types of FL PCP probes with high accuracy and sensitivity.

In the general detection procedure, the as-made PCPs have to be ground into fine powder and dispersed in a specific solvent to form a comparatively stable fluorescence emulsion. Then, the fluorescence detection is carried out by adding the analytes into the fluorescence emulsion and then the change of the FL intensity is recorded by a FL spectrophotometer [14,15]. As we know, most PCPs are insoluble in common solvents, which unavoidably results in the inadequate dispersion for the subsequent sensing experiments. As a result, under such an inhomogeneous system, the non-uniformity of the particle size, the unavoidable PCP particle movements, etc., are inevitable interferences which would affect the accuracy of the fluorescence detection. Therefore, it would be much more desirable to develop soluble FL PCPs that could work under a homogeneous circumstance.

On the other hand, chemical sensors designed for molecules involved in safety and health issues with high sensitivity and selectivity are of great importance in the field of materials science. Carbon disulfide (CS_2_) is an important chemical in the preparation of viscose rayon fibers, but as a volatile organic solvent, it also can become a neurotoxin. It could trigger atherosclerosis and coronary artery disease by exposure at an extended concentration level [16,17,18]. In addition, the nitroaromatic compounds have been widely utilized in varied areas. For example, nitrobenzene is a chemical specifically used in medicine while those bearing di- or tri-nitro groups are well known as high explosives. However, they can also be notorious environmental pollutants causing serious health and safety problems [19,20]. Thus far, the most regular way to detect CS_2_ and nitroaromatic molecules is based on sophisticated analytical instruments and well-trained canines with professional skills [21,22], which might hinder its wide applications, especially in undeveloped areas. Therefore, developing sensitive and selective fluorescence sensors towards these analytes would be a significant but a challenging task.

**Figure 1:**
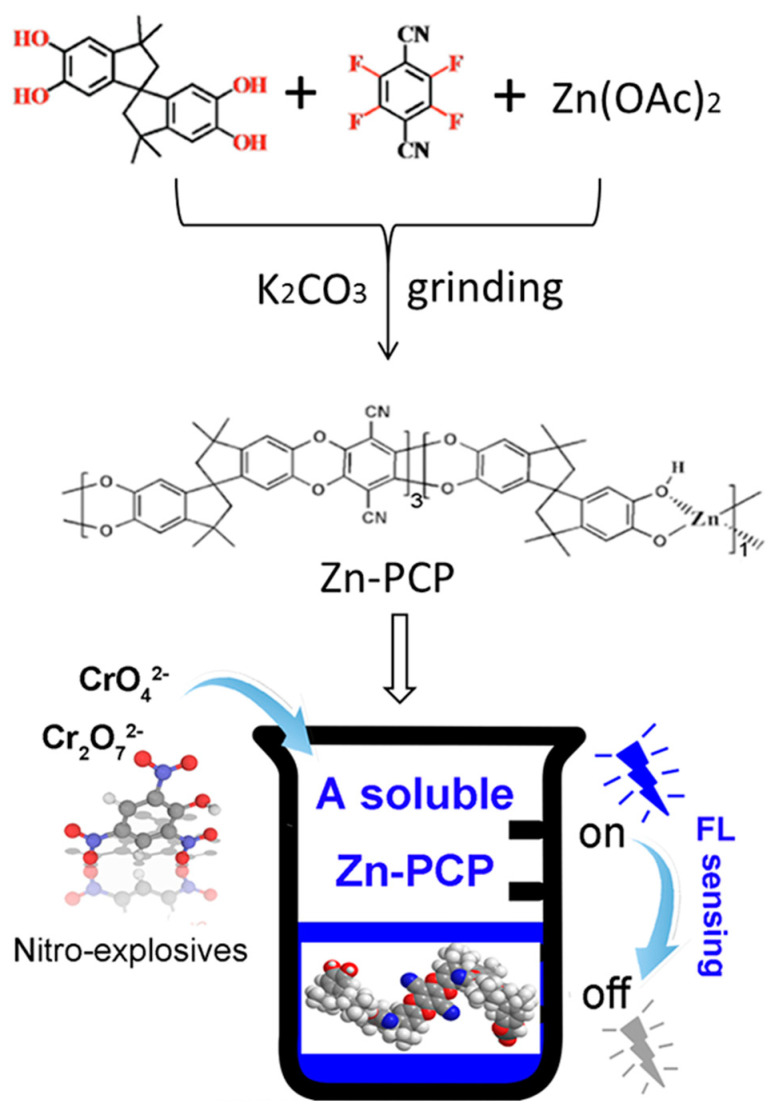
Schematic illustration of synthesis of the soluble Zn-PCP with fluorescence sensing performances towards explosives and toxic anions.

Bearing these in mind, a Zn-PCP is presented in this work which could be soluble in organic solvents, e.g., N,N′-dimethyl formamide (DMF), dichloromethane (CH_2_Cl_2_), and 1,4-dioxane [23,24]. Interestingly, Zn-PCP exhibits an aggregation induced quenching fluorescence after dissolving in solvents. The clear solution emits a bright blue light after being diluted to a low concentration. The emissive solution shows a sensitive fluorescence quenching effect towards nitro-bearing molecules, including but not limited to nitrobenzene, nitroaniline, and nitrophenol. Moreover, it also shows a sensitive fluorescence quenching performance towards dichromate and dichromate ions, demonstrating its detecting ability for toxic metal ion species, Figure 1. As far as we are aware, the fluorescence detection in a homogeneous system using a soluble FL PCP has never been reported up to date.

## 2. Experimental Details

### 2.1. Materials and Methods

All reagents and chemicals were purchased from commercial sources and used without further purification. The UV-Vis absorption spectra were measured at room temperature using a Perkin-Elmer Lambda 900 spectrometer. Powder X-ray diffraction (PXRD) patterns were collected in the angular range of 2*θ* = 3–60° on a Miniflex II diffractometer using CuKα radiation. Fluorescence spectra of the compounds in the solid state and FL sensing experiments were recorded on a PerkinElmer LS55 FL spectrometer at 298 K. Fourier transform infrared (FTIR) spectra were recorded on a Vertex70 IR spectrometer at 298 K. Energy dispersive spectroscopy (EDS) and the scanning electron microscope (SEM) were conducted using a JEOL JSM-6700F scanning electron microscope.

### 2.2. Syntheses of Zn-PCP

The synthesis was carried out according to the procedures in the former article with a slight modification [23,24]. In a typical synthesis, 5,5′,6,6′-tetrahydroxy-3,3,3′,3′-tetramethyl-1,1′-spirobisindane (TTSBI) (0.375 mmol), tetrafluoroterephthalonitrile (0.4 mmol), Zn(OAc)_2_ salt (0.125 mmol), and K_2_CO_3_ (300 mg) were mixed and ground in a mortar over 30 min, and the resulting mixture was washed throughout with deionized H_2_O and methanol (Figure 1). The final powder of Zn-PCP was dried at 333 K for 12 h. The as-made sample could be soluble in CH_2_Cl_2_, DMF, or 1,4-dioxane to afford clear green solution.

### 2.3. Fluorescence Sensing Measurements

The Zn-PCP dissolved in different solvents was prepared by adding 1 mL DMF solution of Zn-PCP (1 mg/mL) into 1 mL organic solvents; for fluorescence sensing, 2 mg Zn-PCP was dissolved in 2 mL DMF and the solution was placed in a quartz cell of 1 cm width, and then a pipette was used to add 10^−2^ M nitrobenzene, nitro-molecule ethanol solution, o-nitrophenol, o-nitroaniline, 10^−3^ M 2,4-dinitroaniline, and TNP or 10^−2^ M Cr-bearing anion solutions into the Zn-PCP solution to record the fluorescence signal. For all the detection measurements, the fluorescence solutions of Zn-PCP were excited at 425 nm while monitoring the corresponding emission wavelengths from 450 nm to 650 nm.

## 3. Results and Discussions

**Figure 2:**
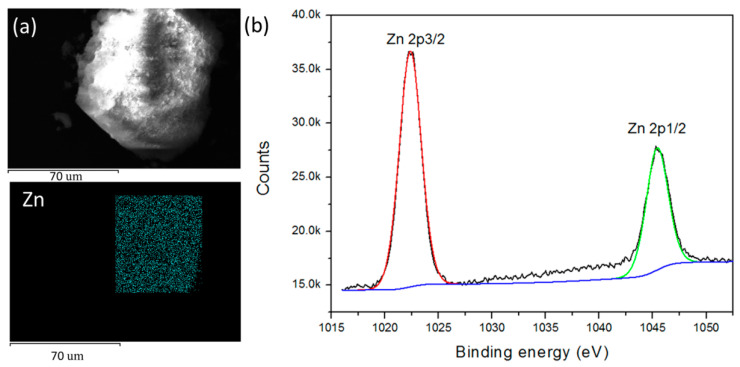
(**a**) SEM image and elemental maps of the as-made Zn-PCP, scale bar is 70 μm. (**b**) XPS measurement for Zn2p in Zn-PCP.

**Figure 3:**
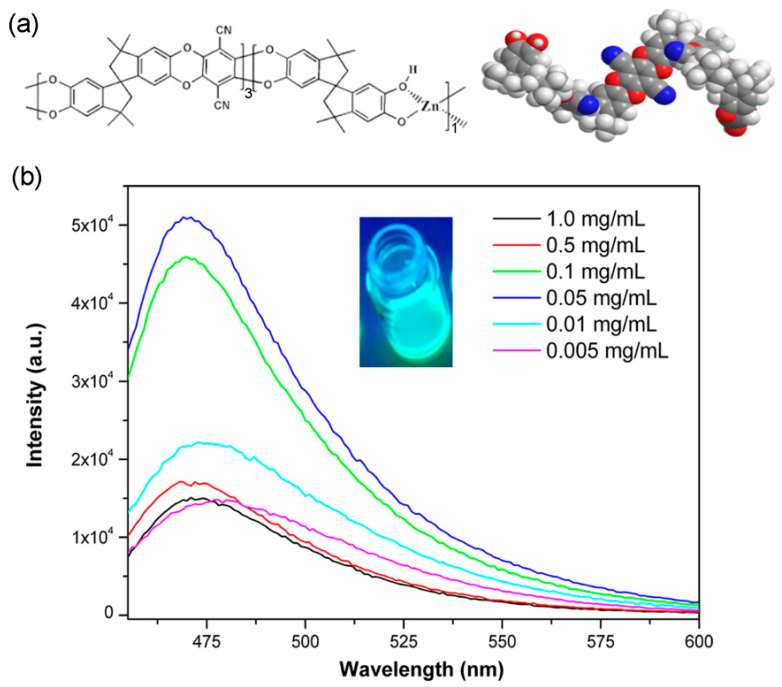
(**a**) The schematic structure for the Zn-PCP. Color scheme: red ball, O; blue ball, N; gray ball, C; light gray ball, H [23,24]. (**b**) The fluorescence spectra of Zn-PCP in DMF solution with different concentrations. Insert is the fluorescence photograph of the Zn-PCP solution under 420 nm light.

The Zn-PCP was prepared through the solid-state interaction of TTSBI ligand bearing -OH groups, tetrafluoroterephthalonitrile and Zn^2+^ ion in a solid state under a base condition (Figure 1) [23,24]. To certify the successfully synthesis of the Zn-PCP, many measurements including but not limited to X-ray photoelectron spectroscopy (XPS), energy dispersive spectroscopy (EDS), the scanning electron microscope (SEM), and Fourier transform infrared (FTIR) spectra have been conducted. As shown in Figure 2a, the SEM measurement demonstrated the as-made Zn-PCP sample was made up of interconnected and aggregated particles with μm sizes. No K and F elements were detected by EDS measurement for the as-made sample, indicating F was removed through coordination reaction while the residual K_2_CO_3_ was clearly removed by washing the samples, Figure S1. The Zn, C, N, O components were further identified by elemental mapping images, revealing specifically Zn element was distributed uniformly in the as-made Zn-PCP particle (Figure 2a and Figure S2). The XPS was applied to analyze the surface chemistry of the as-made Zn-PCP (Figure 2b and Figure S3). As depicted in Figure 2b, there are two energy bands maximized at 1022.8 and 1045.9 eV, which are typical values ascribed to the Zn 2p 3/2 and 2p 1/2 electrons of Zn^2+^ ions. The successful coordination between the ligands and Zn^2+^ ions could be identified by the sharp decrease of -OH vibrational peak around 3300 cm- in FTIR spectra for the as-made Zn-PCP sample (Figure S4) [23]. However, PXRD of the as-made sample indicates Zn-PCP exhibits the amorphous character, which is in accordance with the former research (Figure S5) [23]. The unique folding conformation of TTSBI was transferred to the as-made Zn-PCP through coordination polymerization, resulting in the porous structure by stacking of the zigzag polymer chains (Figure 3a) [23]. The as-made Zn-PCP is soluble in solvents such as DMF, CH_2_Cl_2_, and 1,4-dioxane, which is in accordance with the former research (inset in Figure 3b) [24]. Zn-PCP exhibits no fluorescence at the solid state, but it could emit a bright blue emission maximized at 475 nm after dissolving into DMF. The FL intensity shows some extent of increase by diluting the concentration from 1 to 0.05 mg/mL, and then the FL intensity begins to decrease, exhibiting an aggregation-induced quenching fluorescence (Figure 3b). The chromaticity coordinate for the sample of 0.05 mg/mL is (0.15, 0.21) when monitored at 425 nm excitation wavelength (Figures S6 and S7). The fluorescence of the TTSBI and tetrafluoroterephthalonitrile are also measured to investigate the origin of the luminescence for Zn-PCP. As seen in Figures S8 and S9, the TTSBI exhibits fluorescence with a monochromatic band maximized at 430 nm excited by 360 nm light, while the tetrafluoroterephthalonitrile shows a green emission with two bands maximized at 470 and 540 nm excited with 420 nm light, respectively. Therefore, the origin of the fluorescence would arise from the organic ligands centered (LC) luminescence. The single emission band of the as-made Zn-PCP shows some extent of blue and red-shift compared with that of the TTSBI and the etrafluoroterephthalonitrile ligands. The emission shift might be caused by the energy transfer between the metal ions and linkers after being assembled into the framework materials [1,2,3].

**Figure 4:**
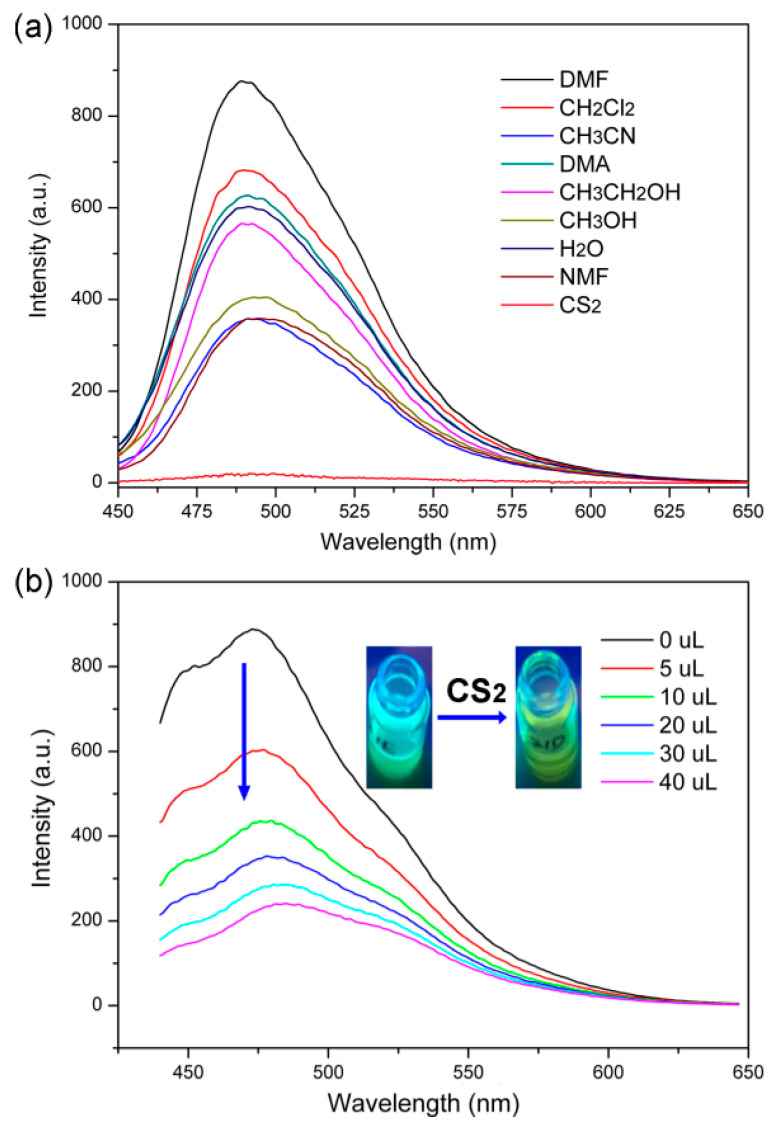
(**a**) The fluorescence of the Zn-PCP dissolved in different solvents. (**b**) The fluorescence spectra of Zn-PCP in DMF solution upon addition of various amounts of CS_2_. Insert is the photograph of fluorescence solutions before and after adding CS_2_.

Then, we investigated the potential fluorescence sensing applications of the Zn-PCP solution. As shown in Figure 4a, the common lab solvents have almost no influence on the fluorescence of Zn-PCP solution except for some extent of changes in their fluorescence intensity. Impressively, it shows an obvious quenching response by adding CS_2_ into the fluorescence solution, demonstrating a selective sensing performance of Zn-PCP towards CS_2_. As we know, although CS_2_ has been widely used in industrial applications, the bad smell and potential leaking risk also could cause environmental and health issues [25,26]. Therefore, compared with the conventional physicochemical detecting methods usually suffering from expensive instruments, professional technicians, and hard popularization in undeveloped areas [27,28,29], it is desirable to explore a more convenient and economic fluorescence sensing way based on FL materials. The detection sensitivity for CS_2_ is further investigated in detail. As depicted in Figure 4b, more than 60% fluorescence intensity is quenched after only 10 μL CS_2_ is being added into the emulsion with only a volume ratio of 0.5 vol% (the volume percentage of CS_2_ in 2 mL Zn-PCP solution, inset of Figure 4b). The sensing sensitivity is comparable to that of Mg_5_(OH)_2_(BTEC)_2_(H_2_O)_4_·11H_2_O (H_4_BTEC = 1,2,4,5-benzenetetracarboxylic acid) which could quench 66% of its fluorescence by adding 10 uL CS_2_ into the FL emulsion [30], but it is much more sensitive than that of other PCP sensors such as Zr_6_(*µ*_3_-O)_4_(OH)_8_(tcbpe)_2_(H_2_O)_4_ (H_4_tcbpe = 4′,4‴,4′′′′′,4′′′′′′′-(ethene-1,1,2,2-tetrayl) tetrakis(([1,10-biphenyl]-4-carboxylic acid)) and [Mg_2_Zn_2_(OH)_2_(1,4-NDC)_3_(H_2_O)_2_]·6H_2_O (1,4-H_2_NDC = 1,4-naphthalene dicarboxylic acid) which can quench more than 60% FL intensity of the emulsions at a concentration of 1.0 vol% and 1.5 vol% CS_2_, respectively [31,32]. According to the former research involving both experimental measurements and DFT calculation results, the interactions occurring between the CS_2_ and the organic linkers in Zn-PCP with the C-H bonds might cause the electron transfer process and quench the fluorescence [32].

**Figure 5:**
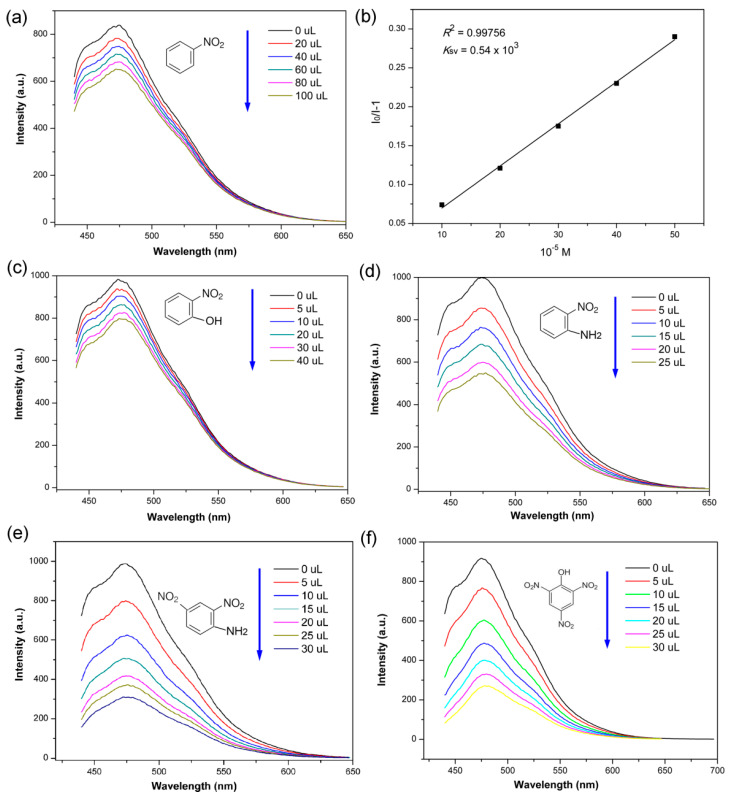
(**a**) The fluorescence spectra of Zn-PCP solution upon addition of various amounts of 10^−2^ M nitrobenzene. (**b**) The corresponding SV equation curve with *K*_sv_ and *R*^2^ values. (**c**–**f**) are the fluorescence spectra of Zn-PCP solution upon addition of various amounts of 10^−2^ M *o*-nitrophenol, *o*-nitroaniline, and 10^−3^ M 2,4-dinitroaniline and TNP.

To our knowledge, nitro-bearing compounds are of great importance to industries and militaries. However, their distinct characters, especially the explosive and toxic characters, also pose a threat to life safety and national security [33,34]. Impressively, the fluorescence solution of Zn-PCP exhibits very sensitive fluorescence quenching behaviour to nitro-bearing compounds. As seen in Figure 5a, as with the sensing of CS_2_, the fluorescence intensity of the Zn-PCP is reduced gradually by adding a 10^−2^ M ethanol solution of the nitrobenzene. To quantitatively analyse the quenching effect, the SV equation of I_0_/I = 1 + *K*_sv_[M] (I_0_ and I are the luminescence intensity of the fluorescence solution without and with addition of quenching molecule, and [M] is the molarity of quencher and *K*_sv_ is the quenching constant) is analysed. As shown in Figure 5b, the SV plot displays a very good linear behaviour with *R*^2^ = 0.99756, and the *K*_sv_ value is calculated to be 0.54 × 10^3^ M^−^. The detection limit value is 1.60 × 10^−1^ M calculated from 3*δ*/slope. On basis of the same fluorescence detecting procedure, the sensing experiments of Zn-PCP towards o-nitrophenol and o-nitroaniline are also performed. As seen in Figure 5c,d, the Zn-PCP also exhibits sensitive fluorescence quenching performances after addition of the 10^−2^ M o-nitrophenol and o-nitroaniline into the FL Zn-PCP solution. The SV plots also display a good linear behaviour with high *R*^2^ values. Accordingly, the *K*_sv_ values are calculated to be 0.106 × 10^4^ and 0.685 × 10^4^ M^−^, respectively (Figures S10 and S11). The detection limit values are 8.17 × 10^−2^ and 1.26 × 10^−2^ M, respectively. The comparative and sensitive quenching results demonstrate the universal sensing performances of Zn-PCP towards nitro-molecules. The sensing performance towards 2,4-dinitroaniline exhibits much more sensitivity than that for the nitro-quenchers with a mono-nitro group. As depicted in Figure 5e, the fluorescence could be quenched more than 60% when only 10 μM 2,4-dinitroaniline is added into the fluorescence solution, and the SV plots show a good linear relationship with *R*^2^ of 0.99475 and the *K*_sv_ is calculated to be 0.15 × 10^5^ M^−^ (Figure S12). The detection limit value is 5.78 × 10^−3^ M. Based on the *K*_sv_ value for comparative study, the sensing performance towards 2,4-dinitroaniline is comparable to that of the porous Mg_5_(OH)_2_(BTEC)_2_(H_2_O)_4_·11H_2_O with *K*_sv_ value of 2.35 × 10^4^ M^−^ [30], demonstrating the potential applications in selective fluorescence detecting for nitro-bearing explosives.

As we know, the trinitrophenol (TNP) is a classic representative of explosive molecules. Here, the fluorescence sensing experiment to TNP is also performed using the same experimental procedures. As shown in Figure 5f, the fluorescence Zn-PCP solution exhibits the most obviously quenching behavior towards TNP, and the fluorescence intensity could be quenched more than 50% when only 7.5 μM TNP is added into the fluorescence solution. The *R*^2^ = 0.9915 indicates the accuracy and sensitive sensing performance. The *K*_sv_ value of 0.16 × 10^6^ M^−^ is two magnitudes of enhanced sensing sensitivity than that of o-nitrophenol and o-nitroaniline (Figure S13). The detection limit value is 5.42 × 10^−4^ M. The reported PCPs for sensing of TNP are listed in Table 1 for a comprehensive study. As shown in Table 1, using *K*_sv_ value as a reference, the TNP detection sensitivity of Zn-PCP is comparable and even better than those of other fluorescence PCP sensors. These PCPs are regularly constructed based on transition or rare earth metal ions, which have to be ground into powder and dispersed in solvents for conducting fluorescence sensing measurements [35,36,37,38,39,40,41,42,43,44,45,46,47,48,49,50].

**Figure 6:**
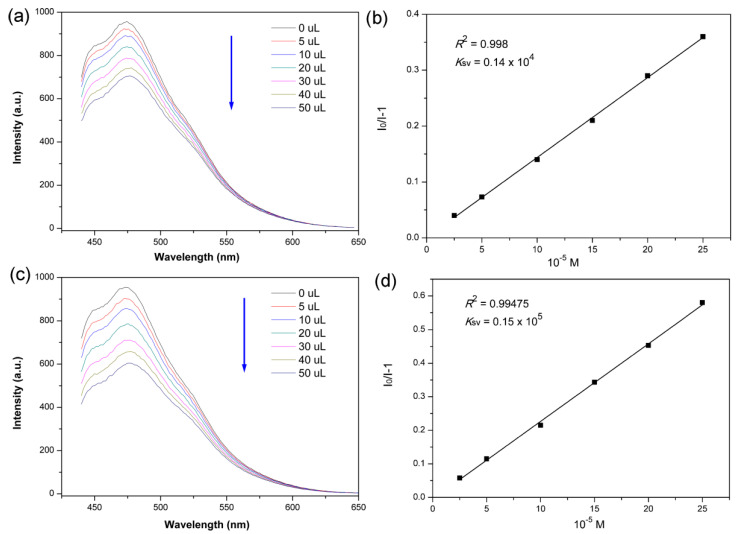
(**a**,**c**) are the fluorescence spectra of Zn-PCP in DMF solution upon addition of various amounts of 10^−2^ M CrO_4_^2−^ and Cr_2_O_7_^2−^ solutions, respectively. (**b**,**d**) are the corresponding SV equation curve with *K*_sv_ and *R*^2^ values.

The Zn-PCP not only exhibits a sensitive detecting response to explosive molecules but also shows a fluorescence quenching performance to toxic metal ion species. As we know, the excess Cr^3+^, especially Cr^6+^ ions, can lead to deformity, and Cr-containing wastes have become a serious environmental issue [51,52]. Therefore, finding a sensitive chemical sensor towards Cr-bearing pollutants is of great significance to probe the environment and health problems. As depicted in Figure 6a,c, the fluorescence intensity of Zn-PCP solution could be significantly decreased gradually by adding 10^−2^ M solutions with CrO_4_^2−^ and Cr_2_O_7_^2−^ anions that acted as fluorescence quenchers. With the addition of CrO_4_^2−^ into the FL Zn-PCP solution in an incremental way, the fluorescence intensity quenches obviously, and the quenching percentage exhibits a good linear relationship with the concentration of quencher, Figure 6a. The linear SV plot displays a high *R*^2^ value of 0.998, and the *K*_sv_ value is 0.14 × 10^4^ M^−^, Figure 6b. While for sensing of Cr_2_O_7_^2−^ anions, the fluorescence intensity of Zn-PCP could be quenched more than 50% when adding only 50 μL quencher, indicating a sensitive sensing performance to Cr_2_O_7_^2−^, Figure 6c. As shown in Figure 6d, the SV plot also exhibits an excellent probing accuracy with an *R*^2^ value of 0.99475 and the *K*_sv_ value is calculated to be 0.15 × 10^5^ M^−^. The detection limit values for CrO_4_^2−^ and Cr_2_O_7_^2−^ are 6.19 × 10^−2^ and 5.78 × 10^−3^ M, respectively. The probing sensitivity could be comparable to the porous transition or rare earth metal-based CP sensors [53,54,55]. Both the sensitive behaviors and the sensing accuracy demonstrate the advantages of using soluble PCP as a fluorescence sensor in probing dangerous and toxic species under a homogeneous environment.

As we are aware, due to the presence of low-lying unoccupied π* molecular orbitals, the nitroaromatic molecules are generally accepted to be efficient acceptors for electrons from fluorophores in their excited state, resulting in the fluorescence quenching response [56,57,58,59]. The sensing sensitivity in detecting nitro-bearing molecules follows the orders of *K*_sv_ values of mono- (o-nitrophenol, o-nitroaniline with 10^4^ M^−^ magnitude) < dual- (2,4-dinitroaniline with 10^5^ M^−^ magnitude) < tri- (TNP with 10^6^ M^−^ magnitude), experimentally demonstrating the nitro group with strong electron withdrawing stability is an efficient acceptor causing the fluorescence quenching. In addition, taking TNP for an instance, the absorption of 10^−3^ M TNP exhibits some extent of overlap with the fluorescence spectra of the Zn-PCP solution. This measurement indicates the energy transfer happens between TNP and Zn-PCP, which further contributes to quenching of the fluorescence with more sensitive detecting performance to TNP than that of other explosives (Figure S14). As the Cr^6+^ metal ion owns unfulfilled d configuration, both CrO_4_^2−^ and Cr_2_O_7_^2−^ anions have strong electron-withdrawing ability as that happening in sensing explosive molecules [60,61,62,63]. The electrons of Zn-PCP excited by photons could be transferred to the anions resulting in a decrease of fluorescence intensity.

## 4. Conclusions

In summary, a Zn-PCP assembled by mechanical grinding of Zn^2+^, TTSBI, and the etrafluoroterephthalonitrile ligands is presented. Different from the former reported PCP materials, the as-made Zn-PCP could be soluble in regularly used lab solvents of DMF, CH_2_Cl_2_, and 1,4-dioxane to form a clear solution which showed an interesting aggregation-induced quenching fluorescence. Importantly, the Zn-PCP could be adopted as a fluorescence sensor to detect nitro-bearing molecules and toxic anions under a homogeneous environment, exhibiting sensitive sensing performances with great accuracy and selectivity. The sensing performances of Zn-PCP were comparatively studied and the potential probing mechanism has also been discussed. This work can not only enrich the sensing members in the family of PCP sensors but also provide a good example for designing novel fluorescence PCP sensors which could be applied under a homogeneous environment.

## Figures and Tables

**Table 1:** The selected CP based fluorescence sensors for TNP.

PCPs	*K*_sv_/M^−1^	Reference
Zn-PCP	0.16 × 10^6^	This work
Cu-CIP	1.07 × 10^4^	[36]
[Eu_3_(L)_3_(HCOO)(μ_3_-OH)_2_(H_2_O)]∙*x*(solv)	2.1 × 10^4^	[37]
Zr_6_O_4_(OH)_6_(L)_6_	2.9 × 10^4^	[38]
[(CH_3_)2NH_2_]_3_[Zn_4_Na(BPTC)_3_]∙4CH_3_OH∙2DMF	3.2 × 10^4^	[39]
[Cd(NDC)_0.5_(PCA)]	3.5 × 10^4^	[40]
[Cd(NDC)L]_2_∙H_2_O	3.7 × 10^4^	[41]
[Zn_8_(ad)_4_(BPDC)_6_O∙2Me_2_NH_2_]	4.6 × 10^4^	[42]
[Zn(BINDI)_0.5_(bpa)_0.5_(H_2_O)]∙4H_2_O	4.9 × 10^4^	[43]
[Zn(BINDI)_0.5_(bpe)]∙3H_2_O	1.29 × 10^4^	
[Zn_3_(TIAB)_2_(IMDC)_2_]·(NO_3_)_2_·(DMF)_2_·(H_2_O)_2_	5.68 × 10^4^	[44]
Zr_6_O_4_(OH)_6_(L)_6_	5.8 × 10^4^	[45]
Zn(NDC)(H_2_O)	6.0 × 10^4^	[46]
Cd(NDC)(H_2_O)	2.38 × 10^4^	
Zn(bipa)(suc)	6.48 × 10^4^	[47]
[Tb(L)_1.5_(H_2_O)]∙3H_2_O	7.47 × 10^4^	[48]
(Me_2_NH_2_)_4_[Eu_4_(DDAC)_3_(HCO_2_)(OH_2_)_2_]·8DMF·9H_2_O	8.6 × 10^4^	[49]
[Zn_4_(DMF)(Ur)_2_(NDC)_4_]	1.08 × 10^5^	[50]
{Mn(Tipp)(A)_2_}*_n_*·2H_2_O	1.18 × 10^5^	[51]

## Data Availability

Data are contained within the article and supplementary materials.

## Supplementary Materials

The following supporting information can be downloaded at: https://www.mdpi.com/article/10.3390/s23249719/s1, Figure S1: The EDX measurement for the as-made Zn-PCP; Figure S2: SEM image and C, N, O elemental maps of the as-made Zn-PCP; Figure S3: The XPS of C1s, N1s, and O1s in Zn-PCP sample; Figure S4: The IR spectrum of the as-made Zn-PCP; Figure S5: The PXRD pattern of the as-made Zn-PCP; Figure S6: The excitation spectra of Zn-PCP solution; Figure S7: The CIE chromaticity diagram for Zn-PCP solution with concentration of 0.05 mg/mL; Figure S8: The excitation and emission spectra of the TTSBI solution; Figure S9: The excitation and emission spectra of the tetrafluoroterephthalonitrile solution; Figure S10–S13: The SV equation curve for *o*-nitrophenol, o-nitroaniline, 2,4-dinitroaniline, and TNP with *K*_sv_ and *R*^2^ values; Figure S14: UV absorption for 10^−3^ M TNP and the FL spectrum of Zn-PCP solution.
