# Supplementary material for: A Soluble Porous Coordination Polymer for Fluorescence Sensing of Explosives and Toxic Anions under Homogeneous Environment
Source: Sensors (Basel). 2023 Dec 9;23(24):9719. doi: 10.3390/s23249719 (PMC10747015; doi:10.3390/s23249719)
Supplement: Supplementary file 1 [file sensors-23-09719-s001.zip › sensors-2667401-supplementary.pdf]

Electronic Supporting Information (ESI) for

A soluble porous coordination polymer for fluorescence sensing of explosives and toxic anions under homogeneous environment

Jiang Jiang [1,#], Zi-Wei Li [2,#], Zhao-Feng Wu [2,*] and Xiao-Ying Huang [2]

[1] Fujian Agriculture and Forestry University, Fuzhou, Fujian, 350002, P.R. China

[2] State Key Laboratory of Structural Chemistry, Fujian Institute of Research on the Structure of Matter, the Chinese Academy of Sciences, Fuzhou, Fujian, 350002, P.R. China

[#] These authors contributed equally to this work.

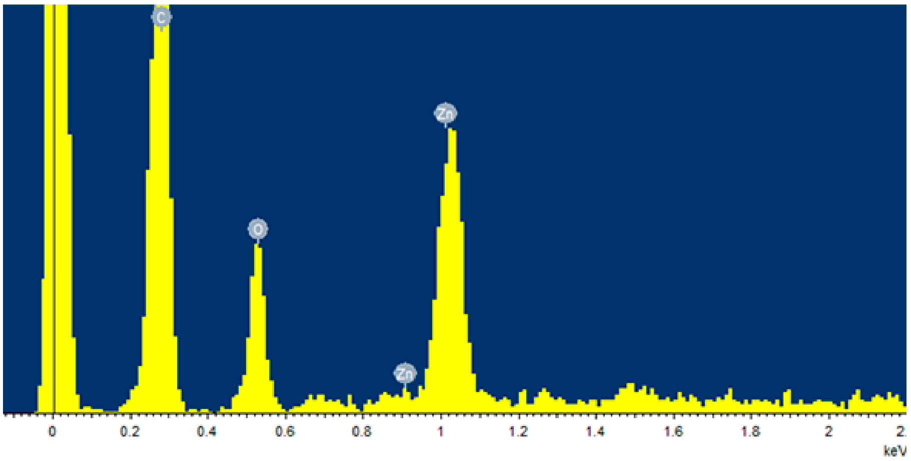

Figure S1. The EDX measurement for the as-made Zn-PCP.

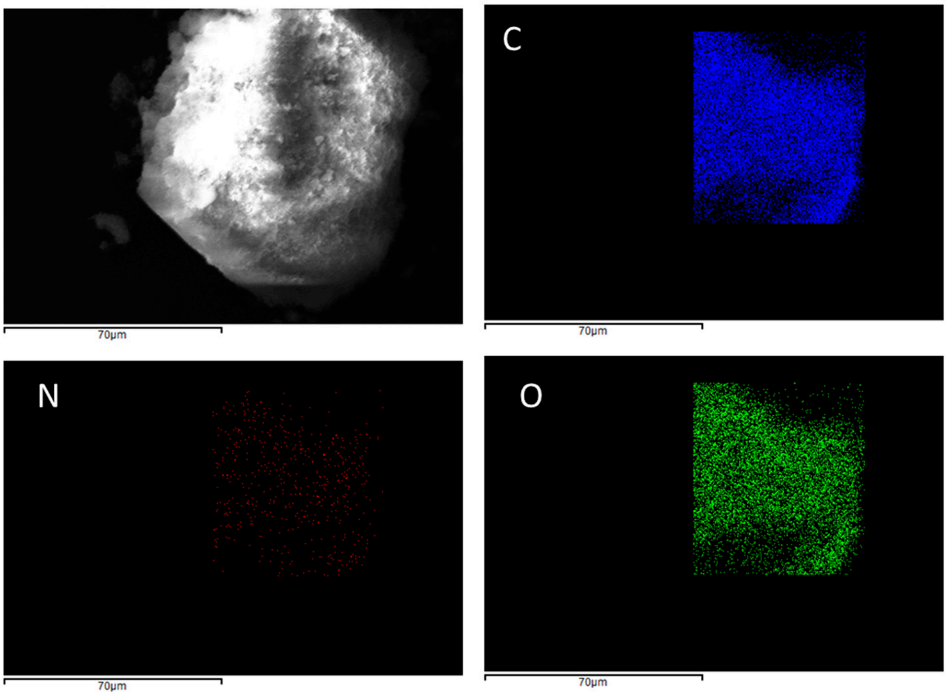

Figure S2. SEM image and C, N, O elemental maps of in as-made Zn-PCP. The scale bar: 70 um.

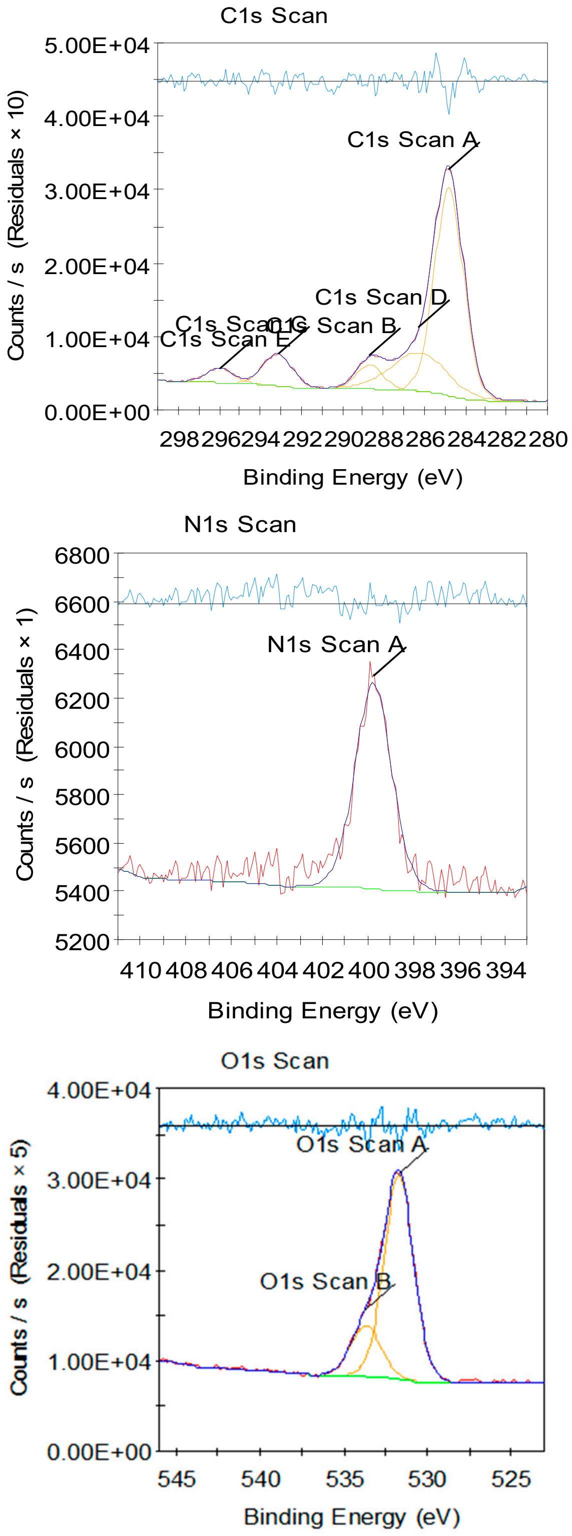

Figure S3. The XPS of C1s, N1s and O1s in Zn-PCP sample.

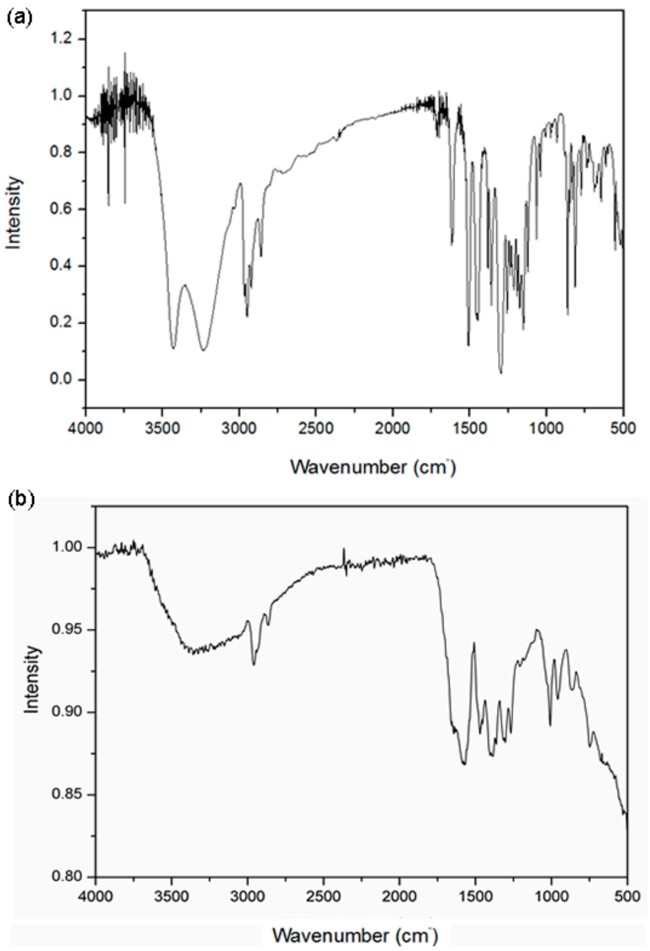

Figure S4. The IR spectrum of the TTSBI ligand (a) and the as-made Zn-PCP (b).

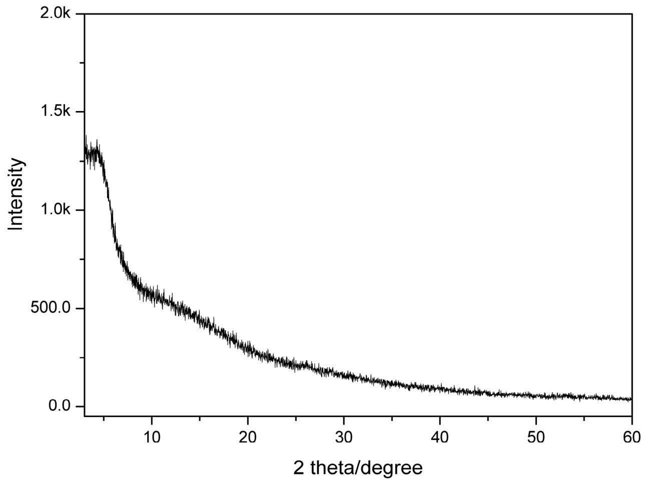

Figure S5. The PXRD pattern of the as-made Zn-PCP.

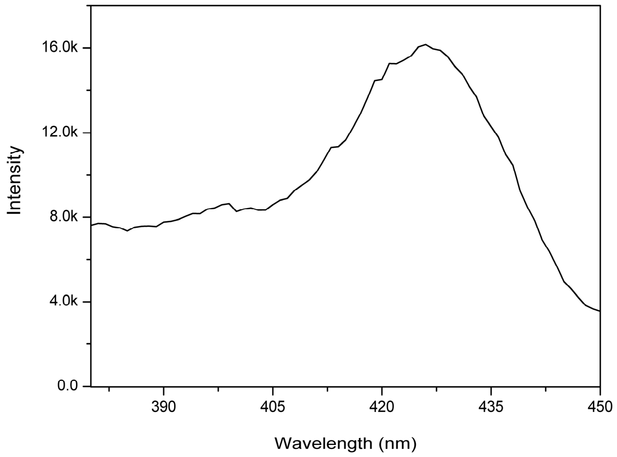

Figure S6. The excitation spectra of Zn-PCP solution.

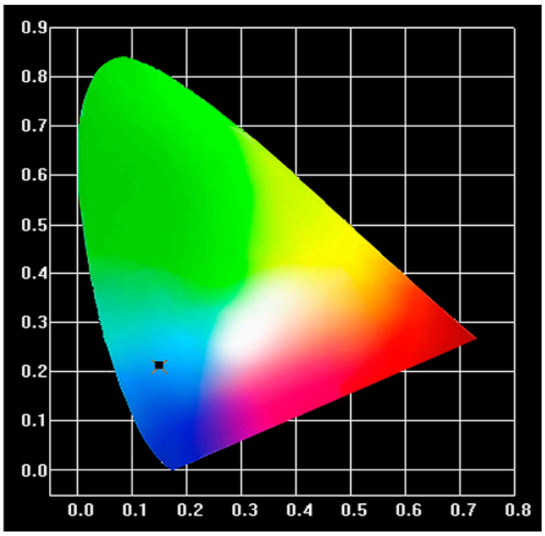

Figure S7. The CIE chromaticity diagram for Zn-PCP solution with concentration of 0.05 mg/mL.

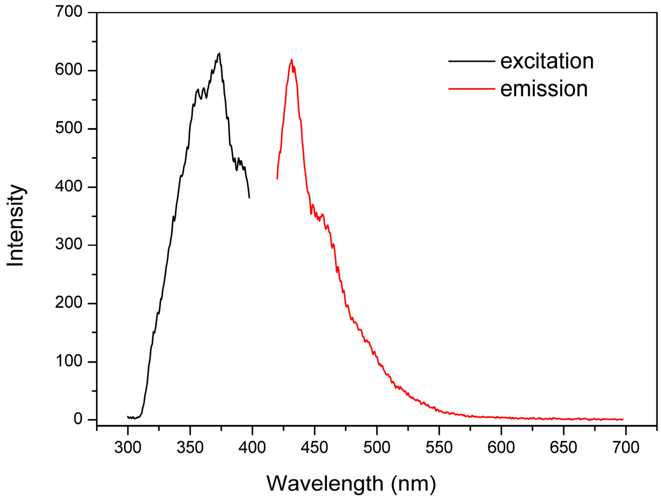

Figure S8. The excitation and emission spectra of the TTSBI solution.

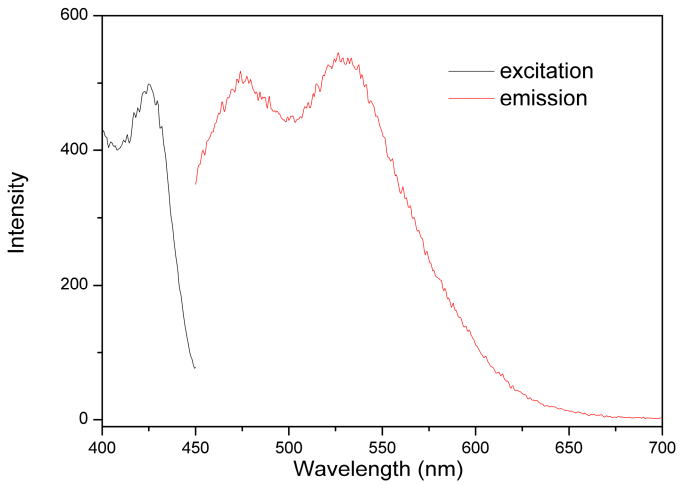

Figure S9. The excitation and emission spectra of the tetrafluoroterephthalonitrile solution.

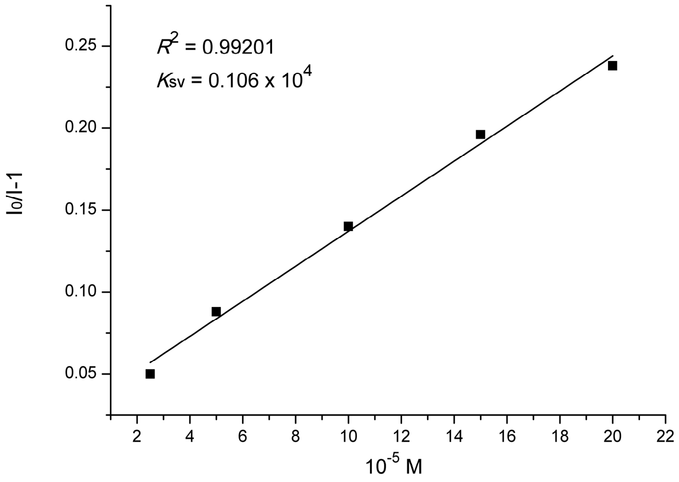

Figure S10. The SV equation curve for *o*-nitrophenol with K_{sv} and R^2 values.

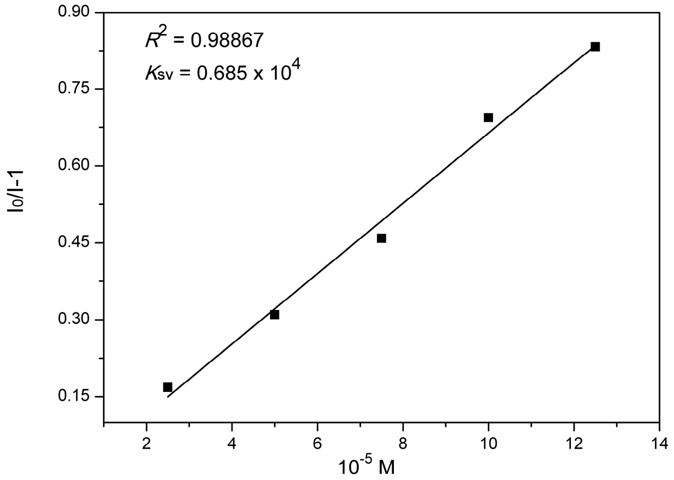

Figure S11. The SV equation curve for *o*-nitroaniline with K_{sv} and R^2 values.

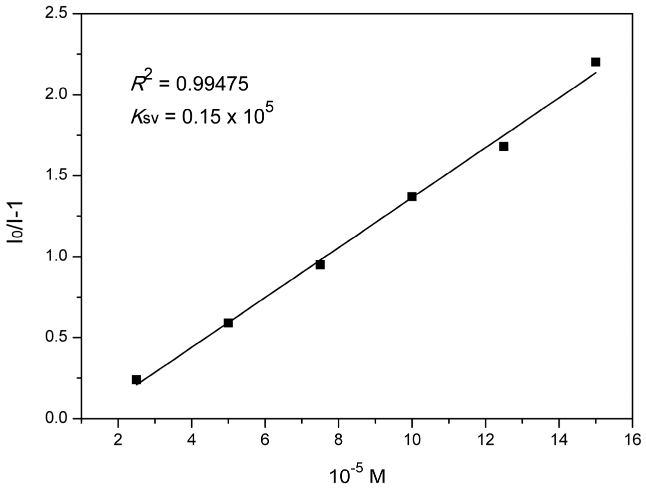

Figure S12. The SV equation curve for 2,4-dinitroaniline with K_{sv} and R^2 values.

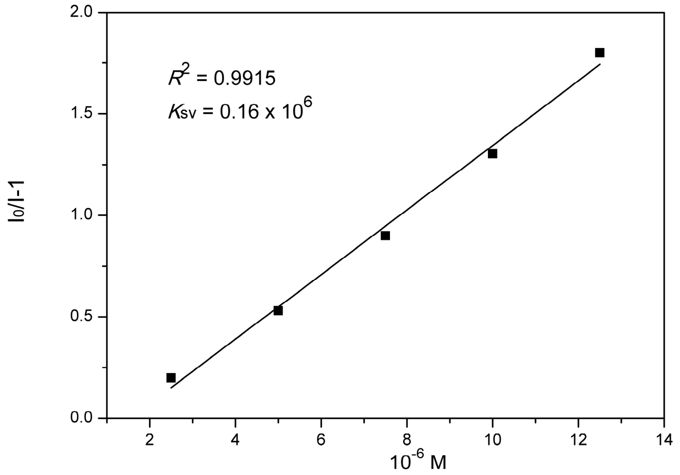

Figure S13. The SV equation curve for TNP with K_{sv} and R^2 values.

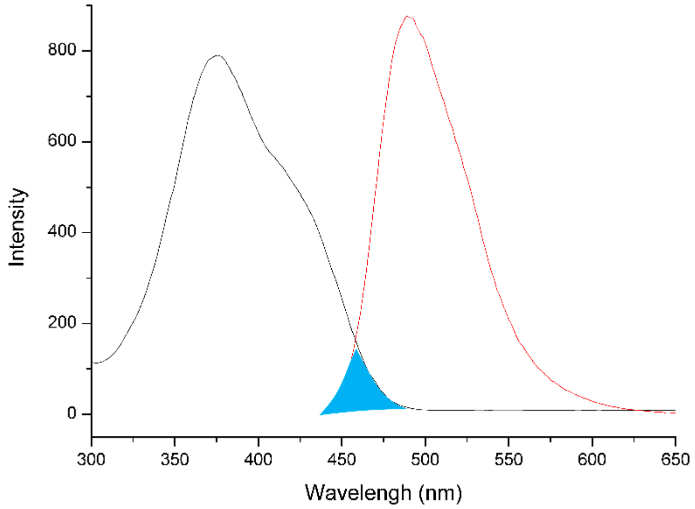

Figure S14. UV absorption for 10^{-3} M TNP (black) and the FL spectrum of Zn-PCP solution (red).
